# The association between glucose dynamics and energy intake in young, healthy women

**DOI:** 10.1038/s41430-026-01727-0

**Published:** 2026-04-04

**Authors:** Zhuoxiu Jin, Alice E. Thackray, Scott A. Willis, James A. King, Jiajin Li, Callum Mould, David J. Stensel

**Affiliations:** 1 National Centre for Sport and Exercise Medicine, School of Sport, Exercise and Health Sciences, Loughborough University, Loughborough, UK; 2 National Institute for Health and Care Research (NIHR) Leicester Biomedical Research Centre, University Hospitals of Leicester National Health Service (NHS) Trust and the University of Leicester, Leicester, UK; 3 Faculty of Sport Sciences, Waseda University, Tokorozawa, Japan; 4 Department of Sports Science and Physical Education, The Chinese University of Hong Kong, Ma Liu Shui, Hong Kong

**Keywords:** Biomarkers, Endocrinology

## Abstract

**Background:**

Glucose has been implicated in the control of appetite and food intake. This study investigated whether glycaemic patterns relate to energy intake and whether appetite-related hormones mediate this relationship.

**Methods:**

Thirty healthy young women (age: 25 ± 4 years; BMI: 21.4 ± 2.0 kg/m^2^) arrived at the laboratory at 8:00 AM after an overnight fast. Upon arrival, participants completed a 30 min resting period, during which the cannula was inserted, and all fasted glucose measurements were collected. Glucose was measured every 5 min using FreeStyle Libre 2™ continuous glucose monitors (CGM), and one venous plasma glucose (VPG) sample was obtained immediately after cannula insertion. Following the 30 min fasting measurement period, participants consumed a fixed breakfast and then remained in the laboratory for an additional 240 min. Glucose was monitored via CGM every 5 min and VPG was measured every 15 min after breakfast. Energy intake was assessed at 240 min using an ad-libitum homogeneous pasta meal. Subjective appetite ratings were collected fasted and every 15 min after breakfast. Appetite-related hormones, including insulin, acylated ghrelin (AG), total glucagon-like peptide-1 (GLP-1) and oxyntomodulin (OXM) were measured in the fasted state, immediately after breakfast (t = 0 min), and subsequently at 30 min intervals after breakfast until t = 240 mins. Menstrual cycle phase was recorded and included as a covariate in all analyses. Associations between glycaemic variables and satiety or energy intake were examined using generalised linear models with a gamma distribution and log link function, adjusting for BMI and menstrual cycle phase. Bootstrap-based causal mediation analyses were conducted to evaluate whether appetite-related hormones mediated any significant associations between glycaemic responses and satiety or energy intake.

**Results:**

CGM-derived glucose nadir (lowest concentration) and dip (deviation of nadir from baseline as a percentage) were significantly associated with subsequent energy intake. A higher glucose nadir was associated with lower energy intake (*β* = -0.17, *p* = 0.003), whereas a greater glucose dip was associated with higher energy intake (*β* = 0.007; *p* < 0.001). When standardised, a one standard deviation (SD) increases in glucose nadir corresponded to an approximately 13% reduction in energy intake, while a one-SD increase in glucose dip corresponded to an approximately 16% increase in energy intake. Glucose nadir (CGM: *β* = 0.23, *p* = 0.041; VPG: *β* = 0.26, *p* = 0.008) and dip (CGM: *β* = -0.01, *p* = 0.006; VPG: *β* = -0.012, *p* < 0.001) derived from both CGM and VPG were also significantly associated with overall satiety measured immediately before the ad libitum lunch. A one-SD increase in glucose nadir was associated with higher satiety (CGM: 20% increase; VPG: 20% increase), whereas a one-SD increase in glucose dip was associated with lower satiety (CGM: 15% reduction; VPG: 17% reduction). Causal mediation analyses provided no evidence that insulin, acylated ghrelin, total GLP-1, or oxyntomodulin mediated these associations (*p* ≥ 0.09).

**Conclusions:**

Glucose dynamics including nadir and dip may influence satiety and subsequent energy intake. These data support for a role of glucose in models of appetite regulation.

## Introduction

Blood glucose concentration has been implicated in the control of eating behaviour [1, 2]. In the 1950s, Mayer formalised the classic glucostatic theory, which proposed that blood glucose utilisation, as determined by arteriovenous glucose differences, may influence energy intake via a mechanism whereby low glucose concentrations and negligible arteriovenous glucose differences are associated with increases in hunger and energy intake [3]. However, subsequent studies have yielded conflicting findings [4–7]. Some research has shown that rapid postprandial declines in blood glucose concentrations after the initial meal-induced peak are associated with increased hunger and meal requests in healthy subjects [4, 5], while other studies have failed to identify meaningful associations of glucose dynamics with appetite sensations and energy intake [6, 7]. These inconsistencies may reflect differences in study design (e.g., observational versus control group comparisons) and the limitations of intermittent blood glucose sampling, typically every 15-30 min, which result in an inability to capture oscillations in glycaemic parameters precisely in response to meal intake [6, 8]. In contrast, the present study addresses this methodological constraint by using continuous glucose monitoring (CGM) to measure interstitial glucose every 5 min, providing a more detailed characterisation of postprandial glucose dynamics.

The development of CGM technology has renewed interest in the relationship between glucose dynamics and energy intake. Two notable studies using CGM have demonstrated that lower postprandial glucose concentrations correlate with higher hunger ratings and energy intake in free-living environments [8, 9]. However, in free-living conditions, actual eating behaviour may not reflect true physiological appetite, as it is constrained by factors such as food availability, dietary options, cost, social influences, environmental context, sleep patterns, and daily routines [10]. In addition, self-reported dietary intake in free-living environment is subject to systematic under-reporting and substantial measurement error [11]. Together, these factors complicate the accurate interpretation of appetite and glucose relationships in free-living settings. Moreover, these studies included both men and women. Emerging evidence suggesting that appetite, energy intake, and responsiveness to metabolic cues may vary across the menstrual cycle in relation to fluctuations in female sex hormones [12, 13]. These limitations highlight the need to clarify the role of glucose dynamics in the regulation of appetite and energy intake in women.

Accumulating research shows that many appetite-related hormones also possess glucoregulatory properties, suggesting that glucose might influence the energy intake by interacting with these hormones. Fluctuations in insulin have been proposed to influence appetite through modulation of hypothalamic glucose-sensing neurons and interactions with other satiety-related peptides [14, 15]. Gut hormones such as acylated ghrelin (AG) also play a pivotal role in signalling energy intake [16, 17]. AG functions through the growth hormone secretagogue-receptor to stimulate appetite and promote food intake [18] and may act as a glucoregulatory signal through modulating insulin/glucagon secretion [19]. Exogenous ghrelin administration can impair glucose tolerance and insulin sensitivity [20, 21]. However, associations between endogenous circulating AG and glucose homoeostasis in observational studies are less consistent, likely due to confounding by adiposity and metabolic status [22]. Furthermore, there is strong evidence for a bidirectional relationship between glucose and AG. Glucose ingestion has been shown to rapidly and markedly suppress circulating AG concentrations in both humans and rodents [23–25], indicating that postprandial glucose dynamics may influence subsequent energy intake partly through modulation of AG. GLP-1 also enhances glucose-dependent insulin secretion and reduces postprandial glycaemic excursions, while also slows gastric emptying and acting on central appetite-regulating pathways to decrease hunger and energy intake [26]. Pharmacological GLP-1 receptor agonists demonstrate these dual actions clearly, producing marked reductions in food intake alongside improved glycaemic control [27]. Studies also have highlighted the role of oxyntomodulin (OXM) in appetite regulation [28, 29]. OXM is co-secreted with GLP-1 postprandially by intestinal L-cells [30] and has been shown to induce weight loss by suppressing appetite [31] and increasing energy expenditure [29]. Oral and intraperitoneal administration of OXM lowers blood glucose concentrations [32, 33]. Therefore, although the original glucostatic theory emphasised direct neuronal sensing of glucose utilisation as the primary determinant of short-term eating behaviour, contemporary models increasingly recognise that glucose fluctuations may also influence appetite via hormonal mechanisms. On this basis, we investigated whether key appetite-related hormones partially mediate the associations between postprandial glucose dynamics and subsequent energy intake.

The primary aims of this study were to examine the relationships between postprandial glucose patterns and subsequent energy intake in a well-controlled laboratory environment; and to investigate the consistency of these relationships when the glycaemic variables are assessed through venous plasma glucose (VPG) compared to CGM. Given that these methods reflect different physiological compartments and may differ in how well they capture glucose signals relevant to eating behaviour, the inclusion of both measures allowed an exploratory comparison of their physiological relevance. A secondary aim of the study was to explore whether the associations between glycaemic variables and energy intake are partly mediated by insulin or appetite-related gut hormones AG, total GLP-1 and OXM.

## Methods

### Participants

Healthy women participated and provided written informed consent. Eligibility criteria were: female, 18-35 years old; body mass index (BMI) between 18.5-24.9 kg∙m^-2^; non-smoker (smoking was considered broadly to include cigarette smoking, vaping, and marijuana use); no known medical conditions, such as metabolic disorders (including diabetes, impaired glucose tolerance and thyroid disease), cardiovascular disease, gastrointestinal disease, or any condition known to affect appetite or glucose metabolism; not taking any medications known to influence CGM accuracy or appetite-related hormone secretion, including acetaminophen (paracetamol), acetylsalicylic acid (aspirin), ascorbic acid (vitamin C), corticosteroids, thyroid medications, or agents affecting glucose regulation or appetite; self-reported habitually consumed three meals daily and no restrictive or controlled eating behaviours, as assessed using the validated three-factor eating questionnaire-R18 [34]; no diagnosed eating disorders; not dieting and weight stable for 3 months before the study ( < 3 kg change); no severe dislike or allergy to any of the study food; and regular menstrual cycle in the past 6 months (oral contraceptive pills, hormonal IUDs, implants, injections, or transdermal patches users excluded). A regular menstrual cycle was defined as a self-reported cycle length between 21 and 35 days [35]. Study procedures were approved by the Loughborough University Ethics Committee (2021-5828-5026) and complied with the Helsinki Declaration guidelines 2013 [36]. Participants were informed of the purpose, procedures, and potential risks before providing written informed consent.

### Study design and procedures

**Fig. 1:**
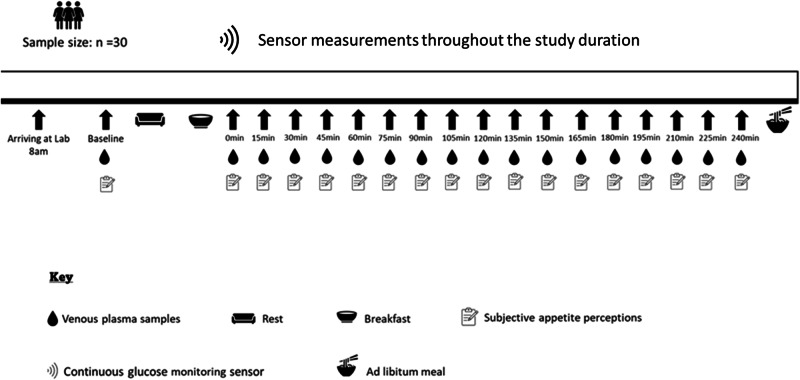
Study protocol.

The study was a single-arm trial consisting of a pre-assessment visit scheduled two days before a main trial to ensure that the CGM operated within its optimal accuracy window during testing. An overview of the protocol is shown in Fig. 1.

During the pre-assessment visit, body mass and stature were measured on an integrated scale and stadiometer with participants in light clothes and without shoes (Seca 285, Hamburg, Germany). Waist and hip circumferences were measured with a nonelastic flexible tape (Seca 201, Hamburg, Germany) while standing. A CGM sensor (Abbott FreeStyle Libre 2^TM^, Witney, UK) was then fitted on the posterior upper arm following the manufacturer’s guidelines. Participants were offered an *ad libitum* homogeneous pasta meal identical to that in the main trial to familiarise them with the meal procedure and ensure acceptability of the food.

In the 24 h before the main trial, participants were required to refrain from alcohol, caffeine, and strenuous exercise. Participants were provided with a Margherita pizza (Goodfella, Green Isle Foods Ltd., Co Kildare, Ireland, 3524 kJ, 44% carbohydrate, 20% protein, 36% fat) in the evening before the main trial and were instructed to consume to fullness, and to avoid eating or drinking anything else except plain water from 10 pm.

On the main trial day, participants arrived at 8 am after fasting for at least 10 h. A cannula (Venflon 20 G/32 mm, BOC Ohmeda, Sweden) was inserted into an antecubital vein on the opposite arm to the CGM sensor. Participants rested for 30 minutes and then consumed a fixed breakfast within 15 min: white bread (100 g), cornflakes (15 g), bananas (150 g with the skin), strawberry jam (15 g), and semi-skimmed milk (200 g) (2121 kJ, 76% carbohydrate, 14% protein, 10% fat). A timer was started once participants finished breakfast (t = 0 min). VPG were measured at baseline (before breakfast), immediately after breakfast (t = 0 min), and every 15 min from t = 0 to 240 min. Plasma insulin, AG, total GLP-1 and OXM concentrations were assessed at baseline, immediately after breakfast (t = 0 min), and every 30 min thereafter until t = 240 min.

Subjective appetite perceptions were measured at baseline and at 15-minute intervals from t = 0 to 240 min using 100 mm visual analogue scales (VAS) [37] to measure ‘hunger’, ‘fullness’, ‘desire to eat’, and ‘prospective food consumption’. Participants remained sedentary throughout the trial. An *ad libitum* lunch was provided 240 min after breakfast, consisting of a homogeneous meal containing fusilli pasta, tomato and basil sauce, and olive oil (72% carbohydrate, 12% protein, 16% fat, 3.6 kJ/ g). Participants were presented with a large serving bowl containing an ample excess of pasta and were instructed to self-serve *ad libitum* and eat until volitional satiation within 30 min. This procedure ensured that participants did not need to request additional food. Avoiding this interaction was intended to minimise potential social-evaluative or monitoring effects [38]. Although additional pasta would have been provided if the initial quantity had been fully consumed, this did not occur in the present study. Energy intake was calculated by weighing the remaining food and subtracting it from the amount initially provided.

### Blood processing and analysis

Glucose concentrations were assessed via CGM and venous blood sampling. A flash glucose monitoring system (FreeStyle Libre2™, Abbott Diabetes Care, Witney, UK) was utilised to measure real-time glucose concentrations, employing the FreeStyle LibreLink app to scan the sensor at baseline, every 5 minutes during the pre-breakfast rest period, and immediately after breakfast until just before the *ad libitum* lunch at 5-minute intervals. Blood samples were collected into pre-chilled 4.9 ml K_3_ EDTA tubes using the Sarstedt S-Monovette® system (Sarstedt, Nümbrecht, Germany). VPG concentrations were measured in duplicate using colourimetric methods on a clinical chemistry analyser (Pentra C400; HORIBA Medical, Montpellier, France) with an intra-assay CV of 0.5%.

Plasma AG and OXM concentrations were measured from blood samples using pre-chilled K_3_ EDTA tubes. For AG assessment, tubes were pre-treated with a 50 μL solution containing p-hydroxymercuribenzoate, potassium phosphate buffer and sodium hydroxide. Samples were centrifuged at 3500 rpm for 15 min at 4°C for plasma separation. For AG, 100 μL of 1 M hydrochloric acid was added to 2 mL of plasma, followed by an additional 5 min centrifugation at the same settings. All samples were stored at −80 °C for later analysis. Enzyme-linked immunosorbent assay kits were utilised to quantify the plasma concentrations of insulin (10-1113-01, Mercodia, Uppsala, Sweden), AG (A05106, Bertin Bioreagent, Montigny-le-Bretonneaux, France), total GLP-1 (EZGLP1T-36K, Merck Millipore, Billerica, USA), and OXM (AL-139, AnshLabs, Wisconsin, USA). The intra-assay coefficients of variation were 6.1% for insulin, 9.3% for AG, 8.3% for total GLP-1, and 5.0% for OXM.

### Study predictors and outcomes

Predictors were defined as follows. Glucose peak and nadir were the highest and lowest postprandial points, respectively. Glucose rise and dip were calculated using the formulas from a previous study [8]: glucose rise = (peak – baseline)/ baseline* 100%; glucose dip = (nadir – baseline)/baseline*100%. To account for variability in baseline glucose concentrations, time-averaged incremental area under the curve (iAUC) was calculated over the specified interval, considering only values above baseline. For AG, the area below baseline was calculated to reflect its characteristic postprandial decline. The overall appetite score was calculated based on subjective appetite perceptions using a previously established formula [39]: overall appetite score = (satiety + fullness + (100 − hunger) + (100 − prospective food consumption)) / 4. The overall appetite score at t = 240 min and time-averaged iAUC of the overall appetite score was assessed and are subsequently referred to as “satiety” or “overall satiety” throughout the manuscript. Energy intake was obtained from the *ad libitum* pasta meal.

### Statistical analysis

The sample size was calculated using the ‘pwr’ package in R [40]. The large free-living CGM study reported an association between glucose dip and energy intake (*r* = -0.27; estimated R² = 7.3%) [8], and a recent meta-analysis reported a standardised mean difference of 0.69 in energy intake between luteal and follicular phases, corresponding to an estimated R² of approximately 10.6% [13]. Given the small sample size and tightly controlled laboratory setting of the present study, it was estimated that glycaemic variables would explain approximately 10% of the variance in energy intake with BMI and menstrual cycle phase together explaining an additional 20%, yielding an anticipated total model R² of 30%. To achieve 80% power, the initial calculation suggested a sample size of 27. To account for potential data loss, this was increased to 30 participants.

Bivariate correlation analysis assessed the association between glucose peak/rise and nadir/dip. The choice between the Pearson or Spearman method was determined by the Shapiro-Wilk normality test results. Associations between postprandial glucose dynamics and both energy intake and satiety were examined using Gamma generalised linear models with a log link and robust standard errors. Each glycaemic variable was included as the primary independent variable, with BMI and menstrual cycle as covariates. Menstrual cycle phase was recorded in 4 categories (menstrual, follicular, ovulation, luteal), which was determined using a calendar-based counting approach method [41, 42]. Participants reported their usual menstrual cycle length, as well as the dates and duration of their most recent menstrual bleeding. Cycle day one was defined as the first day of menstrual bleeding. The cycle day at testing was calculated as the number of days elapsed since cycle day one. Participants were classified as being in the menstrual phase if they were experiencing menstrual bleeding on the day of testing; predicted ovulation was estimated by subtracting 14 days from each participant’s self-reported cycle length. Based on this approach, menstrual cycle phases were defined as follows: follicular (from the first day after bleeding ceased up to two days before the predicted ovulation), ovulatory (a three-day window spanning one day before to one day after the predicted ovulation), and luteal (from two days after the predicted ovulation to the day before the onset of the subsequent menses). In sensitivity analyses, menstrual cycle phase was collapsed into two categories reflecting pre- and post-ovulatory periods: follicular (from cycle day one to the predicted day of ovulation) and luteal (from the day after ovulation to the day before the subsequent menses). The results remained unchanged.

Mediation analysis was performed using a non-parametric bootstrap approach exclusively when a statistically significant association was observed between a glycaemic predictor and the outcome (satiety or energy intake). For each eligible association, insulin, AG, total GLP-1, and OXM were examined as potential mediators using causal mediation analysis with 1000 bootstrap resamples. Path *a* quantified the association between the glycaemic variable and the mediator, path *b* quantified the association between the mediator and the outcome conditional on the glucose variable, path *c* represented the total effect, and path *c*′ represented the direct effect after accounting for the mediator. To accommodate the use of Gamma regression with a log link, zero-valued mediator observations were replaced with a small positive constant equal to half of the smallest observed non-zero value for the corresponding mediator.

In these instances, the model included the significant glycaemic predictor, the outcome, and one appetite-related hormone (Insulin, AG, total GLP-1 or OXM) at a time to assess the potential mediation effects. Data are presented as mean ± standard deviation (SD). Beta-coefficients were back-transformed to express results as fold changes for interpretability along with 95% confidence intervals (CI), and standardised effect sizes expressed as percentage change per one SD increase in glycaemic variables. Statistical significance was set at *p* < 0.05. All analyses and graphs were performed using R version 4.0.5 [43].

## Results

Thirty healthy young women completed the study (age: 26 ± 4 years; BMI: 21.4 ± 2.0 kg/m^2^). CGM readings were unavailable for one participant, resulting in final analyses of 29 participants for CGM and 30 for VPG. Participant characteristics, as well as predictor and outcome variables, are summarised in Table 1. Insulin resistance was estimated using the HOMA-IR, calculated from fasting insulin and VPG concentrations, and these results are presented in Table 1. Fasted and postprandial glycaemic patterns derived from CGM and VPG have been illustrated previously [44].

**Table 1:** Characteristics of participants (*N* = 30 women).

	Mean ± SD or (N)
Age (yrs)	26 ± 4
Ethnicity	Asian (*N* = 23) White (*N* = 4) Arab (N = 2) Latino (*N* = 1)
BMI (kg/m^2^)	21.4 ± 2.0
Waist circumferences(cm)	69.9 ± 5.3
Hip circumferences(cm)	95.7 ± 4.1
Menstrual cycle period	Menstrual (*N* = 6) Follicular (*N* = 14) Ovulatory (*N* = 3) Luteal (*N* = 7)
HOMA-IR	1.2 ± 1.0

Glycaemic Profiles		
	CGM^a^	VPG
Baseline (mmol/L)	4.9 ± 0.7	4.7 ± 0.4
Mean (mmol/L)	6.2 ± 0.8	4.9 ± 0.8
Peak (mmol/L)	8.9 ± 1.4	6.7 ± 0.9
Nadir (mmol/L)	4.4 ± 0.8	3.8 ± 0.7
Rise (%)	85.6 ± 38.9	43.4 ± 22.5
Dip (%)	8.6 ± 19.9	18.8 ± 15.5
Time averaged iAUC (mmol·min/L)	1.3 ± 0.8	0.5 ± 0.6

Appetite-related hormones				
	AG (pg/mL)	Insulin (mU/L)	Total GLP-1 (pmol/L)	OXM (pg/mL)
Baseline	106.0 ± 48.5	5.4 ± 4.2	12.9 ± 7.5	150.4 ± 79.8
prior to lunch (t = 240 min)	133.9 ± 70.0	7.3 ± 5.4	9.6 ± 5.0	188.3 ± 96.4
Peak	136.6 ± 68.1	69.2 ± 40.7	23.9 ± 9.7	292.7 ± 103.2
Rise	0.4 ± 0.4	16.9 ± 19.1	1.2 ± 1.1	3.2 ± 8.6
Nadir	45.4 ± 42.0	6.9 ± 4.7	7.7 ± 4.8	138.3 ± 87.6
Dip	0.6 ± 0.2	-0.6 ± 1.2	0.3 ± 0.3	0.0 ± 0.6
Time averaged iAUC	29.4 ± 18.7	26.4 ± 15.1	3.3 ± 2.6	73.0 ± 75.8

Appetite and Energy intake	
Satiety prior to lunch (t = 240 min)	36 ± 15
Time-averaged satiety iAUC	28 ± 15
EI (kJ)	1832 ± 475

Data are presented as mean ± standard deviation or number of participants. a: CGM-derived data were available for 29 women.*CGM* continuous glucose monitor, *VPG* venous plasma glucose, *iAUC* time-averaged incremental area under the curve, *EI* energy intake. The rise and dip in appetite-related hormones were not expressed as percentages for ease of visual interpretation.

No significant association was observed between CGM-derived glucose peak and nadir (*ρ* = 0.30, *p* = 0.141), whereas a significant positive association was identified between CGM-derived glucose rise and dip (*ρ* = 0.55, *p* = 0.005). For VPG-derived measures, glucose peak was positively associated with nadir (*ρ* = 0.53, *p* = 0.007), while glucose rise was negatively associated with dip (*ρ* = -0.48, *p* = 0.016).

### Primary outcomes: Energy intake and subjective satiety

Postprandial glucose nadir and dip derived from CGM were significantly associated with subsequent EI (Table 2). CGM glucose nadir was inversely associated with EI (*β* = -0.17, *p* = 0.003). Each 1 mmol/L increase in CGM-derived glucose nadir was associated with a 16% lower EI (exp(β) = 0.84, 95% CI: 0.75 to 0.94). When standardised, a one SD increase in glucose nadir corresponded to an approximately 13% reduction in EI, indicating a moderate effect size. In contrast, CGM glucose dip was positively associated with EI (*β* = 0.01, *p* < 0.001, exp(β) = 1.007, 95% CI: 1.005 to 1.010). A one SD increase in glucose dip corresponded to an approximately 16% increase in EI. No significant associations were observed between VPG-derived glucose dynamics and EI (*p* ≥ 0.188, see supplementary Table 1).

**Table 2:** The associations of glucose nadir and dip with energy intake and satiety.

Outcome	Glycaemic variable	exp(β) (95% CI)	% change per 1 SD	p-value
EI	cgm_dip	1.007 (1.005, 1.010)	15.5	< 0.001
EI	cgm_nadir	0.843 (0.754, 0.942)	-12.9	0.003
VAS_240	vpg_dip	0.988 (0.979, 0.996)	-17.4	0.004
VAS_240	vpg_nadir	1.292 (1.070, 1.560)	20.3	0.008
VAS_240	cgm_dip	0.992 (0.986, 0.998)	-15.1	0.006
VAS_240	cgm_nadir	1.255 (1.009, 1.560)	20.2	0.041
VAS_iAUC	cgm_dip	0.988 (0.979, 0.996)	-21.8	0.005
VAS_iAUC	cgm_nadir	1.561 (1.138, 2.142)	43.4	0.006

*EI* energy intake, VAS_240 overall appetite score prior to *ad libitum* lunch (*t* = 240 min). VAS_iAUC incremental area under the curve of the overall appetite score above baseline across lab period; cgm_dip: the glucose dip values from the continuous glucose monitors (CGM); cgm_nadir: glucose nadir from the CGM; vpg_dip: glucose dip values from venous plasma (VPG); vpg_nadir: glucose nadir from VPG.

### Overall satiety score at 240 min (VAS_240)

Both glucose nadir and dip derived from CGM and VPG were associated with overall satiety score at 240 min (VAS_240, Table 2). CGM glucose nadir showed a positive association with VAS_240 (*β* = 0.23, *p* = 0.041, exp(β) = 1.26, 95% CI: 1.01 to 1.56). When standardised, a one SD increase in CGM-derived nadir corresponded to an approximately 20% increase in VAS_240. Similarly, VPG glucose nadir was positively associated with VAS_240 (*β* = 0.26, *p* = 0.008, exp(β) = 1.29, 95% CI: 1.07 to1.56). A one SD increase in VPG nadir corresponded to an approximately 20% increase in VAS_240.

Conversely, glucose dip was inversely associated with VAS_240. CGM glucose dip showed a significant inverse association (*β* = -0.01, *p* = 0.006, exp(β) = 0.99, 95% CI: 0.99 to-1.00). A one SD increase in CGM derived dip was associated with a 15% reduction in satiety at t = 240 min. Similarly, VPG glucose dip was associated with substantially lower VAS_240 scores (*β* = -0.01, *p* < 0.001, exp(β) = 0.99, 95% CI: 0.98 to1.00), and a one SD increase corresponded to an approximately 17% reduction in VAS_240, representing a moderate standardised effect size.

### Satiety incremental AUC (VAS_iAUC)

CGM glucose nadir was positively associated with incremental satiety (β = 0.45, p = 0.006, exp(β) = 1.56, 95% CI: 1.14 to 2.14, Table 2). When standardised, a one SD increase in CGM nadir corresponded to an approximately 43% increase in VAS_iAUC. In contrast, CGM glucose dip was inversely associated with VAS_iAUC (*β* = -0.01, *p* = 0.005, exp(β) = 0.99, 95% CI: 0.98 to 1.00). On a standardised scale, a one SD increase in glucose dip corresponded to an approximately 22% reduction in VAS_iAUC. No significant associations were observed between VPG-derived glucose dynamics and VAS_iAUC (*p* ≥ 0.135, see Supplementary Table 1).

We additionally screened associations with p-values between 0.05 and 0.10 in the presence of large effect sizes ( > 20% change) to assess whether limited statistical power may have obscured potentially meaningful relationships. However, no such associations were observed between energy intake or satiety and other CGM-derived glycaemic metrics, including baseline glucose, mean glucose, peak glucose, or percentage rise from baseline to peak glucose (see Supplementary Table 1).

### Secondary outcomes: mediation analyses

**Fig. 2:**
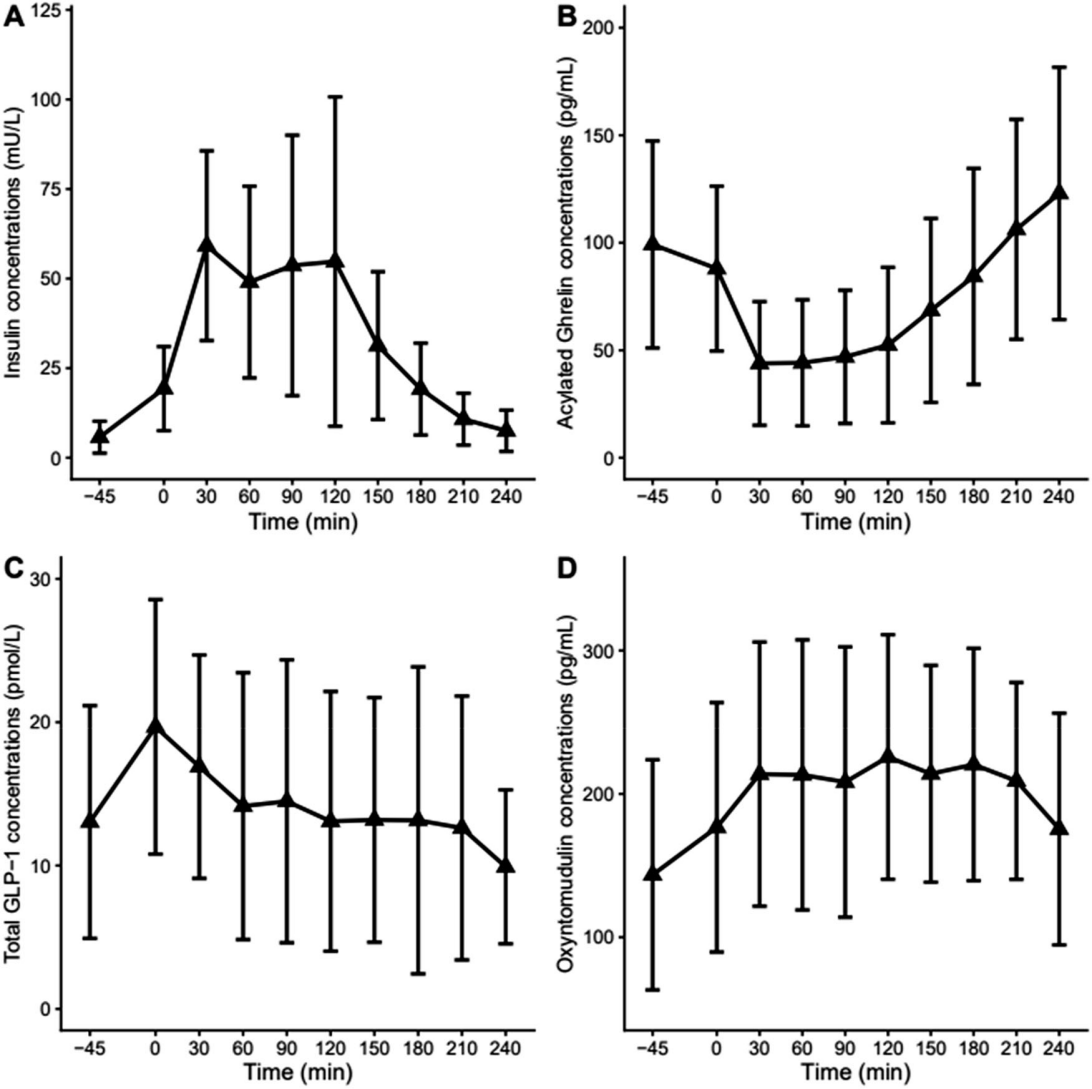
Postprandial profiles of appetite-related hormones over time. Panel **A** shows changes in insulin concentrations; Panel **B** shows changes in acylated ghrelin; Panel **C** shows total glucagon-like peptide-1(GLP-1) concentrations; and panel **D** shows oxyntomodulin changes over time. Data are presented as mean ± standard deviation (SD).

Concentrations of each appetite-related hormone over time are presented in Fig. 2.

Causal mediation analyses provided no evidence that insulin, GLP-1, or OXM mediated the associations between glucose nadir or dip and satiety or energy intake. Across all models, estimated indirect effects (ACME) were small (range:-0.07 to 0.29) and statistically non-significant based on bootstrap inference (*p* ≥ 0.09), while direct effects of glucose nadir or dip on the outcomes remained largely unchanged following inclusion of hormonal mediators. Full path estimates (*a, b, c, and c*′) and mediation results are presented in Supplementary Table 2.

## Discussion

This study explored whether CGM-measured glycaemic dynamics after a fixed meal are associated with subsequent energy intake, and whether the associations are consistent when glycaemic variables are derived from VPG. In addition, this study investigated whether the relationships between glycaemic variables and satiety or energy intake are mediated by insulin or the appetite-related gut hormones AG, total GLP-1, or OXM. The study found that CGM-derived glucose nadir and dip were consistently associated with satiety and subsequent energy intake. In contrast, VPG-derived glucose nadir and dip were associated with overall satiety immediately prior to the ad libitum lunch, but not with incremental satiety or energy intake. Furthermore, neither insulin nor the appetite-related gut hormones mediated the associations between glucose nadir or dip and satiety or energy intake.

Jean Mayer first proposed the link between glucose and appetite, associating low blood glucose utilisation, as indicated by arteriovenous glucose differences, with hunger onset [3]. Research then shifted from absolute arteriovenous glucose differences to postprandial circulating glucose patterns as potential predictors of eating behaviour, with transient glucose declines preceding meal initiation [45] and greater declines correlating with higher energy intake in free-living environments [8, 9]. This study confirms these findings in a controlled laboratory setting, emphasising the role of glucose nadir and dip in predicting appetite and energy intake.

This study also explored other CGM-derived glycaemic variables but found no significant associations with overall appetite or energy intake. Some studies have reported that higher glucose peaks after high glycaemic index meals compared with low glycaemic meals [46] lead to greater energy intake [47]. This could be attributed to the fact that sharp initial rises in blood glucose can lead to greater postprandial dips [48]. Acute glucose rises invoke regulatory processes that lower glucose and contribute to a subsequent drop, potentially below basal concentrations [49]. Greater acute rises and falls in glucose concentrations have been found in meals with more simple carbohydrates [50–52]. In a study [8] which did not find associations between glucose peak/rise and energy intake, participants were offered five different types of meals (i.e., high carbohydrate, high fat, oral glucose tolerance test, UK average, and high-fibre meals) and the averaged glucose profiles from these varied meal types were reported. The meal provided in the present study had a moderate glycaemic index (65 units), which may partly explain why a greater postprandial glucose rise was not followed by a larger subsequent glucose decline. Consistent with this, correlation analyses revealed inverse associations between glucose rise and dip, indicating that a higher glucose peak was associated with a smaller subsequent glucose decrease. One possible explanation for this pattern is heterogeneity in insulin sensitivity among participants, as suggested by variation in HOMA-IR values. In addition, neither mean nor incremental glucose concentrations predicted appetite or energy intake, reinforcing the notion that glucose nadir or dip—rather than absolute glucose levels or overall glycaemic excursions—may be more relevant for short-term appetite regulation and food intake.

While CGM-derived glucose nadir and dip were associated with satiety and energy intake, we did not see the association between VPG-derived nadir or dip and energy intake. This discrepancy likely reflects fundamental physiological and methodological differences between glucose measured in interstitial fluid (CGM) and venous blood (VPG), which represent distinct compartments and may convey different information regarding systemic energy status. In healthy individuals, venous blood samples yield lower postprandial glucose concentrations than arterialised blood during oral glucose tolerance testing in healthy populations [53], reflecting tissue glucose uptake and extraction, whereas CGM aligns well with fingertip and arterialised VPG after a glucose load, though with a 15-minute time lag [54].

Few studies have directly measured glucose nadir or dip in healthy populations. One research correlates fingertip glucose concentrations below baseline in healthy individuals with higher subsequent energy intake [55], and the other reported no differences when glucose remains above baseline [56]. Consistent with these findings, the present study demonstrated that an earlier glucose dip (60-120 min after the first postprandial peak), during which glucose concentrations remained above baseline in most participants, was not associated with subsequent energy intake. In contrast, a later glucose dip that fell below baseline was significantly associated with energy intake (see Supplementary Table 1), supporting the relevance of below-baseline glucose dips in short-term appetite regulation. However, studies using VPG show conflicting results. One study found lower energy intake in healthy individuals whose VPG concentrations returned to baseline, compared to individuals whose VPG concentrations remained below baseline [57].In contrast, the present study was not able to link glucose nadir/dip from VPG with subsequent energy intake, suggesting that arterialised blood samples may better reflect glucose dynamics in healthy populations.

Population differences may further contribute to these inconsistencies. In individuals with obesity, who often exhibit impaired glucose utilisation, no significant differences have been observed between arterial and VPG concentrations [58]. Additionally, in a cohort of individuals with overweight, an earlier VPG-derived glucose nadir was associated with a more rapid return of hunger, despite a similar magnitude of glucose dip extent [59]. Taken together, these findings highlight the importance of considering the glucose measurement compartment when investigating glucose and appetite relationships. Future studies should directly compare glucose nadir and dip derived from arterialised blood, interstitial fluid, and venous plasma within the same individuals, and across different metabolic populations, to clarify which signals most accurately reflect central energy sensing and appetite regulation.

The present study found no evidence that insulin or appetite-related gut hormones including AG, total GLP-1 or OXM mediated the associations between postprandial glycaemic nadir or glycaemic dip and satiety or energy intake. Although studies have proposed that insulin, rather than glucose per se, is the primary determinant of short-term energy intake [7], our findings do not support this interpretation. One possible explanation is the metabolic heterogeneity within our cohort. Calculation of HOMA-IR (shown in Table 1) indicated that several participants displayed insulin resistance, which may blunt the anorexigenic action of insulin. Experimental and mechanistic studies show that insulin reduces food intake in metabolically healthy, lean individuals, whereas this effect is attenuated in those with overweight, obesity, or insulin resistance due to reduced central insulin sensitivity and impaired insulin transport across the blood-brain barrier [60]. Thus, variability in insulin sensitivity within our sample may have obscured any mediating role of insulin on short-term energy intake.

AG yet in controlled laboratory feeding paradigms its postprandial suppression often correlates only modestly with subsequent energy intake [18, 61]. GLP-1 and OXM are both postprandial satiety peptides; however, their acute appetite-reducing effects in healthy individuals are generally small and highly variable, influenced by metabolic status, gastric emptying and nutrient composition [62–65]. These findings suggest that the hormonal changes observed in this study were not sufficiently large, as in clinical infusion studies [28], to mediate the relationship between postprandial glucose nadir or dip and energy intake. A further methodological consideration is that the mediation analyses examined insulin, AG, GLP-1 and OXM individually, without including multiple hormones in the same model. Appetite regulation is a highly integrated process involving overlapping and partially redundant hormonal signals and analysing one hormone at a time may not fully capture their potential additive or interactive contributions. However, given the small sample size, incorporating multiple hormonal predictors simultaneously would substantially reduce statistical power and increase the risk of model overfitting. Future studies with larger and more diverse samples should consider multivariate or network-based modelling approaches to quantify combined hormonal effects and better characterise the coordinated endocrine response underlying postprandial appetite regulation.

Furthermore, in this study, an alternative explanation is that glucose itself may directly influence the neural regulation of appetite, independent of appetite-related hormones. The brain contains specialised glucose-sensing neurons, particularly in the hypothalamus, that respond to falling circulating glucose concentrations to initiate compensatory eating and prevent hypoglycaemia [66]. These glucose sensors can be activated by peripheral signals transmitted through fenestrated regions of the hypothalamus located outside the blood-brain barrier, enabling rapid detection of postprandial glycaemic declines [67]. Beyond homoeostatic regulation, hedonic processes may also play a role. Meals that elicit a larger acute rise and subsequent fall in circulating glucose have been shown to stimulate the reward and craving centres of the brain [68], potentially driving hunger and subsequent food intake [69]. This may further promote hunger and increase the likelihood of overeating, independent of appetite-related hormones.

Strengths of this study include the formal mediation analysis involving insulin and established appetite-related hormones, AG, total GLP-1, and OXM, as well as the strict standardisation and objective assessment of energy intake within the laboratory setting. The latter helped mitigate the primary limitation - the small sample size, particularly in a single-arm study design. However, the findings should be interpreted in the context of several limitations. Most notably, the sample consisted exclusively of young, healthy women of predominantly Asian ethnicity with BMIs in the normal range. This limits generalisability, as glucose and appetite relationships may differ in older adults, individuals with overweight or obesity, or people with metabolic impairments such as prediabetes and type 2 diabetes, given their altered or impaired the glucose metabolism [70,71]. The study also examined a relatively narrow set of predictors, and it is likely that additional factors contribute to the observed relationships. In addition, the menstrual phase was estimated using a calendar-based counting method, which provides only an approximate indication of cycle stage and is less accurate than biological verification methods, such as urinary luteinising hormone testing or hormonal profiling; these methods should be considered in future studies involving women. Future research could also use randomised controlled designs and incorporate a broader range of dietary stimuli (e.g., meals with varied glycaemic index or macronutrient composition) to test the robustness of glucose-appetite coupling across contexts. Studies involving more diverse populations will also be important for determining whether the patterns observed here extend to other demographic and metabolic profiles.

In conclusion, this study demonstrated that CGM-derived glucose nadir and dip predict satiety and subsequent energy intake, independent of postprandial fluctuations of insulin and other appetite-related gut hormones, i.e., AG, total GLP-1 and OXM. These data further support the role of glucose in appetite regulation models.

## Supplementary information

Supplementary Table 1

Supplementary Table 2

## Data Availability

The data presented in this study are available through the Loughborough University Research Repository at: 10.17028/rd.lboro.31150822.
